# Development and Immunogenicity of a Five-Antigen Strangles Vaccine Based on Equine Ferritin Nanoparticles in Mice

**DOI:** 10.3390/vetsci13060527

**Published:** 2026-05-28

**Authors:** Min Wang, Weiguo Zhang, Rongkuan Sun, Jiafang Nong, Wei Guo, Xiaojun Wang

**Affiliations:** 1 State Key Laboratory for Animal Disease Control and Prevention, Harbin Veterinary Research Institute, Chinese Academy of Agricultural Sciences, Harbin 150069, Chinazweiguo00@163.com (W.Z.);; 2 College of Veterinary Medicine, Nanjing Agricultural University, Nanjing 210095, China; 3 China-Kazakhstan Joint Laboratory for Herbivorous Animal Disease Research, Harbin Veterinary Research Institute, Chinese Academy of Agricultural Sciences, Harbin 150069, China; 4 Institute of Western Agriculture, Chinese Academy of Agricultural Sciences, Changji 831100, China

**Keywords:** equine ferritin, nanoparticle vaccine, strangles, subunit vaccine, adjuvant-free, immune protection, mouse model

## Abstract

As a naturally self-assembling protein nanocarrier, ferritin enables multivalent antigen display and functions as an intrinsic adjuvant to enhance vaccine-induced immune responses. *Streptococcus equi* subsp. *equi (S. equi*) is the causative agent of equine strangles, an acute and highly contagious respiratory disease responsible for substantial economic losses worldwide. However, currently available vaccines often show suboptimal immunogenicity and limited protective efficacy. In this study, we developed a recombinant equine ferritin (rHF)-based nanoparticle vaccine, rSE5Mix, presenting five core protective antigens (EQ8, EQ5, CNE, IdeE, EAG). The fusion proteins efficiently assembled into uniform nanoparticles. Immunization of BALB/c mice elicited rapid, high-titer antigen-specific IgG antibodies and a balanced Th1/Th2 immune response without additional adjuvants. Following lethal challenge with *S. equi*, rSE5Mix-immunized mice showed 100% survival and markedly milder clinical signs. Histopathological analysis demonstrated significantly alleviated organ damage, and bacterial loads in major tissues were reduced to nearly undetectable levels. Importantly, compared with the octavalent tandem vaccine rSE8, rSE5Mix induced faster elevation of partial antigen-specific antibodies, especially for EQ8 and CNE. Their antibody titers were comparable at later stages. This study developed a safe and effective ferritin nanoparticle vaccine candidate against equine strangles and verified that equine ferritin is a promising candidate delivery platform for veterinary bacterial vaccines.

## Simple Summary

Ferritin is a naturally self-assembling protein nanocarrier with strong intrinsic immune adjuvant activity. Equine strangles is a highly contagious respiratory disease affecting horses globally. In this study, we constructed a recombinant equine ferritin nanoparticle vaccine displaying five protective antigens. Without additional adjuvants, this recombinant five-antigen mixed ferritin nanoparticle vaccine rapidly induced high-titer specific antibodies and afforded complete protection against lethal challenge in mice. Relative to the recombinant eight-antigen tandem ferritin vaccine, this five-antigen vaccine elicited a faster early antibody response while achieving comparable titers at later stages. This study validates equine ferritin as a promising veterinary vaccine delivery platform and provides a safe and effective candidate against equine strangles.

## 1. Introduction

Ferritin is a ubiquitous iron-storage protein composed of two distinct subunit types, heavy (H) and light (L) chains. The H subunit possesses ferroxidase activity for iron oxidation, while the L subunit contributes to iron mineralization and structural stability [1]. Structurally, ferritin naturally self-assembles into a spherical hollow cage architecture, which lays a structural foundation for its application as a nanovaccine platform [2]. In physiological solutions, 24 subunits undergo spontaneous assembly into a hollow, cage-like structure measuring approximately 10–12 nm in outer diameter [3]. Owing to its natural nanocarrier properties, ferritin exhibits excellent biocompatibility, high thermal stability, and considerable genetic amenability. Significantly, ferritin functions as an intrinsic immunostimulatory agent, boosting immune responses without requiring external adjuvants [4,5]. Through genetic fusion, heterologous antigens can be presented on the ferritin surface at high density and valency. Such multivalent presentation facilitates recognition and uptake by immune cells [6] and effectively activates both humoral and cell-mediated immunity [7,8,9].

Equine ferritin was the first ferritin to be purified, crystallized, and structurally analyzed [10], laying a foundation for ferritin family research. Early studies focused on its assembly, iron metabolism, and structural features, with few reports on nanovaccine applications [11]. Currently, it is only used in basic biochemical research and has not been developed as a delivery vector for veterinary vaccines [12]. As an endogenous equine protein, it has excellent biocompatibility and safety [13], making it a promising platform for equine vaccine design.

Strangles represents an acute and highly contagious respiratory ailment in equine species that is induced by *S. equi* [14,15]. Affected equids typically present with pyrexia, depression, mucopurulent nasal exudate, and suppurative lymphadenopathy [16]. This infectious disease spreads rapidly among susceptible populations, resulting in compromised growth and reduced productivity [17,18], and, in severe instances, death. These outcomes result in considerable economic losses for the intensive horse industry [19,20,21].

Vaccination remains the primary control measure for strangles. Nevertheless, existing vaccine platforms—such as killed whole-pathogen, modified live, and traditional subunit products—often exhibit weak immunogenicity, suboptimal protective efficacy, and safety concerns [22,23,24]. No commercial strangles vaccine is currently available in China; therefore, head-to-head comparison with commercial vaccines was not performed in this study.

Previous studies by He et al. identified eight protective antigens of *Streptococcus equi*: EQ8, EQ5, CNE, IdeE, EAG, SclF, SclI, and SclC. The eight-component vaccine conferred 100% protection in mice, versus only 67% for the inactivated *S. equi* HLJ2018 vaccine, laying a solid basis for our antigen selection [25]. Excessive antigens hinder tandem expression and protein purification [26]. Thus, five highly immunogenic core antigens were selected to develop nanoparticle vaccines. Novel delivery systems are urgently required to enhance the efficacy and protection of adjuvant-free subunit vaccines [27,28].

Based on the above background, we constructed a nanoparticle vaccine using five immunodominant *S. equi* antigens. This study aims to provide a safe, effective, and novel candidate for the prevention and control of strangles.

## 2. Materials and Methods

### 2.1. Strains, Plasmids, Tissues and Animals

Equine kidney specimens were harvested from healthy horses during necropsy at a local equine farm in Heilongjiang Province. The *S. equi* HLJ2018 strain, along with prokaryotic expression vectors (pGEX-6p-1, pET-30a, and pET28a-*se8*) and five antigen-encoding plasmids (pGEX-6p-1-*eq8*, pGEX-6p-1-*eq5*, pGEX-6p-1-*cne*, pGEX-6p-1-*ideE*, and pGEX-6p-1-*eag*), were acquired from the Innovation Laboratory of Equine Infectious Diseases and Lentiviruses, Harbin Veterinary Research Institute, Chinese Academy of Agricultural Sciences. Four-week-old female specific pathogen-free (SPF) BALB/c mice were acquired from Changsheng Biotechnology Co., Ltd., Shenyang, China. All animal husbandry procedures and experimental treatments were performed in strict accordance with the internationally recognized Guide for the Care and Use of Laboratory Animals. Animals were kept under standard barrier conditions at 22 ± 2 °C with 50 ± 10% relative humidity and a 12:12 h light–dark cycle.

### 2.2. Ethical Approval

All procedures involving animals strictly followed the Laboratory Animal Care and Use Guidelines endorsed by the Ministry of Agriculture of China. The study protocol was reviewed and approved by the Animal Ethics Committee of Harbin Veterinary Research Institute, Chinese Academy of Agricultural Sciences (approval number: IACUC-250311-01-GR, approved on 11 March 2025).

All mice were euthanized by cervical dislocation under isoflurane anesthesia at the end of the experiment or when reaching humane endpoints, and death was confirmed by cessation of heartbeat and respiration.

### 2.3. Purification of rHF

The gene sequence for equine ferritin was obtained from the NCBI GenBank database under accession number XM_070228902. Specific primers for *HF* gene amplification were designed as follows, *HF*-F: 5′-ATGCCGATGGTTCTGCCGCT-3′, *HF*-R: 5′-TCAGCTTCTGCATCTGGGTG-3′. The HF gene is 546 bp in length. Equine kidney tissue samples were processed for total RNA isolation using TRIzol^®^ reagent (Invitrogen, Carlsbad, CA, USA, Cat. No. 15596018) according to the manufacturer’s instructions. The extracted RNA was reverse-transcribed into complementary DNA (cDNA) using the PrimeScript™ RT Reagent Kit (TaKaRa, Dalian, China, C-at. No. R037A). The target equine ferritin (HF) gene was amplified by PCR using the above cDNA as template with PrimeSTAR^®^ Max DNA Polymerase (TaKaRa, Dalian, China, Cat. No. R045A). The PCR thermal cycling program was set as: 98 °C for 3 min; 35 cycles of 98 °C for 10 s, 58 °C for 15 s, 72 °C for 30 s; final extension at 72 °C for 5 min; hold at 4 °C.

Two recombinant plasmids, pET-*HF* and pGEX-*HF*, were constructed by inserting the HF gene into prokaryotic vectors pET-30a and pGEX-6p-1, respectively, and the accuracy of the constructs was verified via Sanger sequencing commissioned to Jiansu Biotechnology Co., Ltd., Harbin, China.

The confirmed plasmids were transformed into Escherichia coli BL21 (DE3) competent cells via heat shock. Single positive colonies were selected and cultured in LB medium containing kanamycin or ampicillin (based on vector resistance) at 37 °C with shaking at 220 rpm until the OD_600_ reached 0.6–0.8. Protein expression was initiated using 0.5 mM IPTG at 16 °C for 15 h. The induced bacterial cells were collected by centrifugation at 8000 rpm for 10 min. Lysis buffer was prepared strictly according to the instructions of the Beyotime protein purification kit. The bacterial pellet was resuspended in lysis buffer (pH 7.4), and then lysed by ultrasonication in an ice-water bath with a SCIENTZ-IID ultrasonic cell disruptor (Scientz Biotechnology Co., Ltd., Ningbo, China). The ultrasonic parameters were set as 300 W power, 3 s working time/5 s interval, with a total operation duration of 20 min.

The lysate was centrifuged at 12,000 rpm for 20 min at 4 °C to obtain the supernatant containing soluble target protein. The recombinant HF protein with His-tag was purified using BeyoGold™ His-tag Protein Purification Kit (Beyotime Biotechnology, Shanghai, China, Cat. No. P2226). The GST-tagged rHF was purified using BeyoGold™ GST-tag Protein Purification Kit (Beyotime Biotechnology, Shanghai, China, Cat. No. P2262), all affinity chromatography columns were purchased from Beyotime Biotechnology (Shanghai, China, Cat. No. FCL12). The eluted protein was dialyzed against PBS (pH 7.4) at 4 °C for 12 h with 3 buffer changes, then concentrated using Amicon Ultra-15 centrifugal filter units (Merck Millipore, Darmstadt, Germany, Cat. No. UFC910096) with a 10 kDa molecular weight cutoff (MWCO). The protein samples were separated by sodium dodecyl sulfate–polyacrylamide gel electrophoresis (SDS-PAGE) and visualized via Coomassie brilliant blue staining (KRT, Tianjin, China, Cat No. ab.001.10) [29]. Western blotting was performed on the purified rHF using His-tag-specific antibody. The primary antibody was 6 × His Tag McAb (Proteintech, Rosemont, IL, USA, Cat No. 66005-1-Ig) diluted at 1:5000, and the secondary antibody was CoraLite647 Goat Anti-Mouse IgG (H + L) (Proteintech, Rosemont, IL, USA, Cat No. SA00001-1) diluted at 1:10,000.

### 2.4. Preparation of rSE5Mix Vaccine

Five fusion genes, including *eq8-HF*, *eq5-HF*, *cne-HF*, *idee-HF* and *eag-HF*, were amplified using five laboratory-preserved plasmids (pGEX-6p-1-*eq8*, pGEX-6p-1-*eq5*, pGEX-6p-1-*cne*, pGEX-6p-1-*idee* and pGEX-6p-1-*eag*) as templates to obtain *eq8*, *eq5*, *cne*, *idee* and *eag* gene fragments, respectively. Meanwhile, the *HF* gene was amplified using horse kidney tissue cDNA as the template. To avoid steric hindrance between antigens and ferritin that affects correct folding and assembly of fusion proteins, a flexible linker peptide was introduced between the C-terminus of each antigen gene and the N-terminus of the *HF* gene. The amino acid sequence of the linker was GGGGSGGGGS, with the corresponding nucleotide sequence: 5′-GGCGGTGGCGGTAGCGGCGGTGGCGGTAGC-3′.

Splicing by overlap extension PCR (SOE-PCR) was employed to assemble each antigen gene with the HF gene into a complete fusion gene. The primers used are listed in Table 1.

The fusion gene fragments and linearized pET28a vector (digested by restriction enzymes) were added into the recombination system. Homologous recombination was performed using the Seven seamless cloning kit at 37 °C for 1 h. The recombinant products were transformed into *Escherichia coli* DH5α competent cells and spread on kanamycin-resistant LB agar plates. Positive clones carrying the corresponding fusion genes were screened by colony PCR. After amplification culture in LB liquid medium, plasmid DNA was extracted and verified by Sanger sequencing at Harbin JianSu Biotechnology Co., Ltd. (Harbin, China). After confirming the correct sequence, the recombinant expression plasmids were preserved, namely *pET28a-eq8-HF*, *pET28a-eq5-HF*, *pET28a-cne-HF*, *pET28a-idee-HF* and *pET28a-eag-HF*.

Under optimized induction and purification conditions (including suitable IPTG concentration, induction temperature and time, and affinity chromatography parameters), the five target recombinant fusion proteins were expressed and purified with high purity. Before vaccine preparation, each purified recombinant protein was quantified using a BCA protein assay kit (Thermo Fisher Scientific, Rockford, IL, USA, Cat No. 23225). The five dialyzed and purified recombinant fusion proteins were adjusted to a concentration of 1 mg/mL with PBS and the five proteins were mixed at a precise equimolar ratio (1:1:1:1:1) to prepare the multivalent recombinant vaccine designated rSE5Mix [30]. Each 1 mL of rSE5Mix consisted of 170 μg rEQ8-HF, 257 μg rEQ5-HF, 209 μg rCNE-HF, 214 μg rIdeE-HF, and 150 μg rEAG-HF (all at 1 mg/mL).

### 2.5. Identification of Five Recombinant Proteins

The five fusion proteins were divided into two parallel groups for Western blotting analysis. The primary antibody was 6 × His Tag McAb (Proteintech, Rosemont, IL, USA, Cat No. 66005-1-Ig) diluted at 1:5000, and the secondary antibody was CoraLite647 Goat Anti-Mouse IgG (H + L) (Proteintech, Rosemont, IL, USA, Cat No. SA00001-1) diluted at 1:10,000. In the second group, the primary antibody was *Streptococcus equi*-positive equine serum, which was collected from horses naturally infected with strangles at a horse farm in Heilongjiang Province by the Equine Infectious Disease and Lentivirus Research Team of Harbin Veterinary Research Institute, with a dilution ratio of 1:500. The secondary antibody was Rabbit anti-Equine IgG (H + L) Secondary Antibody (Novus, Centennial, CO, USA, Cat No. NB120-6700FR) diluted at 1:10,000. SDS-PAGE was used for protein separation, and the separated proteins were electrotransferred to PVDF membranes. Membranes were blocked and then incubated with primary antibodies overnight at 4 °C. After thorough washing with TBST, the membranes were incubated with fluorescent secondary antibodies at room temperature. Following another round of TBST washing, the membranes were directly scanned and imaged using an Odyssey infrared imaging system.

The five recombinant fusion proteins with concentration of 1 mg/mL, and then delivered to the Electron Microscopy Laboratory of Harbin Veterinary Research Institute for transmission electron microscopy (TEM) observation. Meanwhile, the hydrodynamic particle size and polydispersity index (PDI) of the samples were determined using a BeNano 90 Zeta nanoparticle size and Zeta potential analyzer (Malvern Panalytical, Malvern, UK). The samples were filtered through a 0.22 μm membrane to remove aggregate impurities before detection. All measurements were performed at room temperature and repeated three times for each sample to analyze the particle size distribution and homogeneity of the five recombinant proteins.

### 2.6. Challenge Model and Immunoprotective Assay Against rSE5Mix

A murine challenge model previously established by our laboratory was adopted in this study. The model was constructed using BALB/c mice infected with *Streptococcus equi* subsp. *equi* strain HLJ2018, and its minimum lethal dose (MLD) was determined as 10^5^ CFU via preliminary experiments. For subsequent challenge assays, 10 × MLD (10^6^ CFU) was used, which was referenced from previous related studies [25] and verified by preliminary experiments in this study. This dose induced 100% mortality in the PBS control group, confirming it as a stable and effective lethal dose that sufficiently ensures the discriminatory ability of the challenge model for evaluating vaccine protective efficacy. Thirty healthy BALB/c mice were randomly assigned into three groups via a random number table: rSE5Mix, rHF and PBS control group. A single-blind method was adopted throughout the bacterial challenge and subsequent sample detection procedures.

Mice in the rSE5Mix group were intraperitoneally injected with 100 μL of pentavalent vaccine formulation containing 17 μg rEQ8-HF, 25.7 μg rEQ5-HF, 20.9 μg rCNE-HF, 21.4 μg rIdeE-HF and 15 μg rEAG-HF. The rHF group received 100 μg of purified HF protein, while the control group was administered 100 μL of sterile PBS. All mice were primed on day 0 and boosted on day 14. On day 28 after primary immunization, all animals were intraperitoneally challenged with 200 μL of bacterial suspension containing 10-fold MLD of *S. equi* subsp. *equi* HLJ2018.

Clinical signs and survival rates were monitored throughout the experiment. Peripheral blood samples were collected on days 0, 7, 14, 21, 28, 35, 42, 49 and 56 to measure specific antibody titers. All surviving mice were humanely euthanized at the termination of the experiment.

To assess the in vivo toxicity and tolerability of recombinant proteins, body weight was dynamically recorded during the trial. Baseline body weight was measured on day 0 prior to immunization, and weekly weighing was subsequently performed at a fixed time using an electronic balance with a precision of 0.01 g. In accordance with animal welfare and ethical guidelines, a body weight loss of ≥20% or severe clinical manifestations including lethargy, abnormal behavior and ruffled fur was defined as the ethical endpoint. Mice reaching this endpoint were immediately euthanized. Body weight data were statistically analyzed, and the biosafety of recombinant proteins was comprehensively evaluated combined with clinical observations.

**Figure 1:**
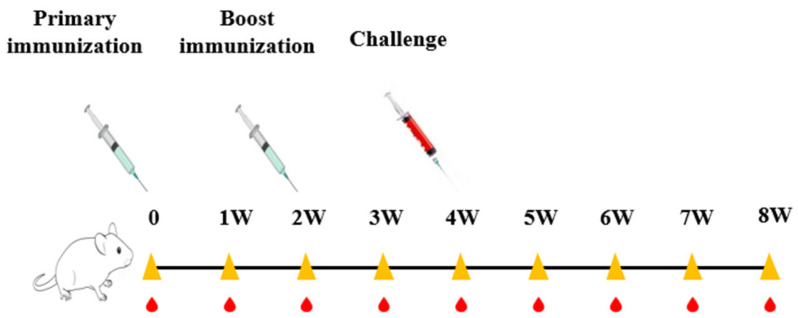
Schedule of rSE5Mix and rHF immunization and *S. equi* challenge.

The detailed experimental schedule is shown in Figure 1.

The survival and clinical signs of mice were monitored daily for 14 consecutive days.

### 2.7. Detection of rSE5-Specific Antibody Levels by Indirect ELISA

GST-tagged EQ8, EQ5, CNE, IdeE and EAG proteins prepared (Figure S3) were used as coating antigens. All antigens were diluted to 1 ng/μL with sterile PBS, added to 96-well plates at 100 μL per well, and incubated overnight at 4 °C. The plates were washed three times with PBST (200 μL per well, shaken for 5 min each time), then blocked with 5% skimmed milk at 37 °C for 2 h.

Mouse serum samples were two-fold serially diluted starting at 1:200 and added at 100 μL per well. Meanwhile, 24 samples of *S. equi*-negative mouse serum were set as negative controls, with three replicate wells for each sample, followed by incubation at 37 °C for 1 h. After washing, HRP-conjugated goat anti-mouse IgG (Bioss, Beijing, China, Cat. No. bs-40296G-HRP) diluted at 1:10,000 was added at 150 μL per well and incubated at 37 °C for 30 min.

Subsequently, 80 μL of TMB substrate (Solarbio, Beijing, China, Cat No. PR1200-100mL) was added for 10 min of light-shielded color development, and the reaction was terminated with 60 μL of 2 M H_2_SO_4_. The absorbance at 450 nm was detected by a microplate reader within 15 min. The positive threshold was defined as the mean OD value of negative sera plus three standard deviations. The highest dilution showing a positive result was regarded as the antibody titer. All assays were performed with three technical replicates and two biological replicates.

### 2.8. Immunological Comparison Assay of rSE5Mix and rSE8

BALB/c mice were randomly allocated into three groups (rSE5Mix, rSE8, and PBS control), with 10 mice in each group.

Detailed protocols for the preparation and dosage of rSE5Mix are provided in Section 2.6. Owing to the self-adjuvanting activity of equine ferritin nanoparticles, rSE5Mix was administered without additional adjuvants. The rSE8 formulation was emulsified with commercial Alhydrogel^®^ adjuvant (InvivoGen, Toulouse, France, Cat No. vac-alu) at a 1:1 volume ratio. Each mouse received 100 μg of rSE8 fusion protein (1 mg/mL, 100 μL per dose), following the dosage reported by He et al. [25]. Mice in the PBS group were given 100 μL of sterile PBS. All three groups were immunized intraperitoneally with an identical injection volume of 100 μL per mouse, and the immunization procedure followed the protocol described in Section 2.6.

### 2.9. Detection of rSE5 and rSE8-Specific Antibody Levels by Indirect ELISA

The procedure was performed as described in Section 2.7.

### 2.10. Cytokine Detection by Means of qRT-PCR

The RT-qPCR assay adopted TB Green^®^ Premix Ex Taq™ II (Tli RNaseH Plus) (Catalog No. RR820A) supplied by Takara Biomedical Technology Co., Ltd., Beijing, China. The kit was packaged at 2 × 1.25 mL, consisting of premixed enzyme solution, TB Green fluorescent dye, optimized PCR buffer and RNase-free water.

At 24 h after bacterial challenge, three mice from each group were randomly selected and euthanized. Spleen tissues were collected and immediately frozen in liquid nitrogen for RNA storage. Total RNA was extracted from spleen samples and reverse-transcribed into complementary DNA (cDNA) with a commercial reverse transcription kit. Quantitative real-time PCR (qRT-PCR) was performed using specific primers (listed in Table 2) in a 25 μL reaction system containing 12.5 μL of 2 × TB Green Premix, 1 μL of forward primer, 1 μL of reverse primer, 2 μL of cDNA template, and 8.5 μL of ddH_2_O to detect mRNA expression levels of IL-4, IL-10 and IFN-γ. β-actin was used as the internal reference, and all reactions were performed in triplicate. The relative mRNA expression levels were analyzed to evaluate the cellular immune responses induced by vaccination [31].

### 2.11. Pathological Changes and Organ Bacterial Load Detection After Challenge

Heart, liver, spleen, and lung tissues were aseptically collected from mice that either succumbed to bacterial challenge or were euthanized at the experimental endpoint. Each tissue sample was equally divided into two aliquots. One aliquot was fixed in 4% paraformaldehyde for 24 h and subsequently submitted to the Pathology Laboratory of Harbin Veterinary Research Institute, Chinese Academy of Agricultural Sciences, for the preparation of histopathological sections. Upon receipt of qualified sections, subsequent histopathological observation and analysis were performed. The other aliquot was accurately weighed and homogenized with sterile phosphate-buffered saline (PBS) on ice. Following centrifugation, the supernatant was serially diluted and plated onto Columbia blood agar plates. The plates were incubated at 37 °C for 18–24 h, and colony counts were performed to calculate the tissue bacterial load, which was expressed as colony-forming units per gram of tissue (CFU/g). The limit of detection (LOD) for bacterial quantification in this experiment was 10^2^ CFU/g [25]. 

Statistical analysis of the tissue bacterial loads among the three groups was conducted to evaluate the in vivo bacterial colonization and the protective immune efficacy induced by the vaccine.

### 2.12. Safety Assessment of Recombinant Equine Ferritin (rHF) in Horses

To verify whether recombinant equine ferritin (rHF) could induce autoimmune responses in horses, five healthy horses (No. 1#–5#) were selected and intramuscularly immunized with 200 μg of rHF for primary immunization, followed by a booster injection at a 2-week interval (approval number: IACUC-251230-01-SW, approved on 28 December 2025).

**Figure 2:**
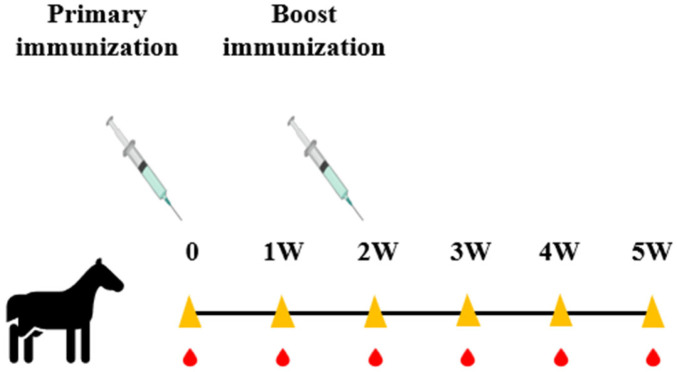
Schedule Immunization diagram for the safety trial of rHF in horses.

The body temperature, appetite and mental status of horses were continuously monitored for 5 days after each immunization, and blood samples were collected weekly. Purified GST-tagged HF protein was used for coating (Figure S3). Indirect ELISA (iELISA) was performed using the serum samples collected at each time point as the primary antibody (diluted 1:400) and HRP-conjugated Goat Anti-Equine IgG (H + L) (SouthernBiotech, Birmingham, AL, USA, Cat. No. 6040-05) as the secondary antibody at a dilution of 1:10,000. The detailed experimental schedule is shown in Figure 2.

### 2.13. Statistical Analysis

All independent experiments were performed in triplicate. All statistical analyses were carried out using GraphPad Prism 8.0. Before statistical testing, the Shapiro–Wilk test was used for normality assessment, and the Brown–Forsythe test was adopted to evaluate homogeneity of variance. Longitudinal repeated-measures data, including body weight dynamics and antibody titers, were analyzed via two-way repeated-measures ANOVA, followed by Tukey’s multiple comparisons test. All data are expressed as mean ± standard deviation (SD), with 95% confidence intervals (CI) presented where applicable. A *p*-value less than 0.05 was regarded as statistically significant. Significance was denoted as: *p* < 0.05, *p* < 0.01, *p* < 0.001; ns indicates no significant difference.

## 3. Results

### 3.1. Purification and Characterization of rHF

**Figure 3:**
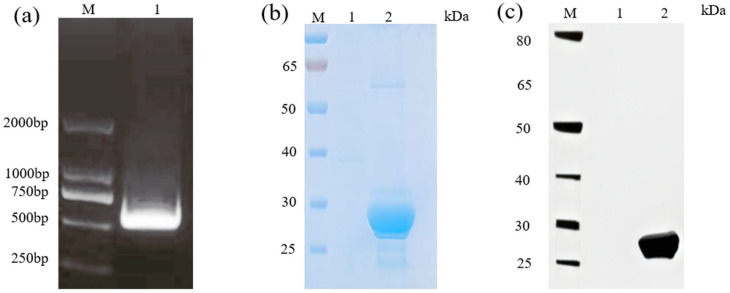
Identification of recombinant equine ferritin (rHF). (**a**) PCR amplification of the HF gene. M: DNA marker; Lane 1: PCR product. (**b**) SDS-PAGE analysis of purified rHF. M: Protein marker; Lane 1: pET30a empty vector (The supernatant of bacterial lysate obtained from the induced expression of the blank pET30a plasmid was mixed with 5 × loading buffer at a volume ratio of 1:4 for preparation, the sample loading volume was 10 μL); Lane 2: Purified rHF (Total protein loading amount: 8 μg, with 2 µL 5 × Loading buffer). (**c**) Western blot analysis of purified rHF. M: Protein marker; Lane 1: Empty vector control; Lane 2: Purified rHF (Total protein loading amount: 8 μg, with 2 µL 5 × LoadingLoading buffer).

According to the equine ferritin sequence (XM_070228902) from NCBI, specific primers were designed to amplify the target gene from equine kidney tissue. Agarose gel electrophoresis showed a specific band around 500 bp, consistent with the expected size of 546 bp. The PCR product was cloned into the pET-30a vector, and the correctly sequenced plasmid was designated pET-30a-HF (Figure 3a). Purified rHF was adjusted to 1 mg/mL and separated by SDS-PAGE and identified via Coomassie brilliant blue staining (KRT, Cat No.: ab.001.10) (Figure 3b). Western blotting was performed using mouse anti-His tag antibody (diluted 1:2000) as the primary antibody and HRP-conjugated goat anti-mouse IgG (diluted 1:10,000) as the secondary antibody. A clear specific band matching the expected size was observed, verifying the identity of rHF (Figure 3c).

### 3.2. Expression and Purification of Five Recombinant Fusion Proteins

**Figure 4:**
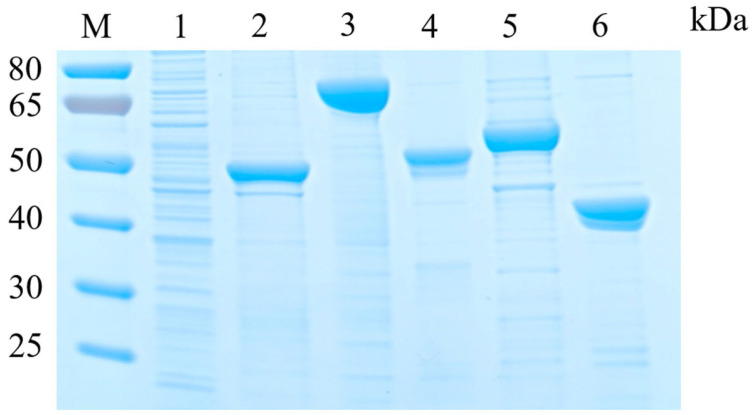
Purification of five target antigen–equine ferritin (HF) recombinant fusion proteins by Ni–NTA affinity chromatography, followed by SDS-PAGE identification and Coomassie brilliant blue staining (KRT, Cat No.: ab.001.10). M: Protein marker. Lane 1: pET28a empty vector (The supernatant of bacterial lysate obtained from the induced expression of the blank pET28a plasmid was mixed with 5 × loading buffer at a volume ratio of 1:4 for preparation, the sample loading volume was 10 μL), Lane 2: EQ8-HF (Total protein loading amount: 8 μg, with 2 µL 5 × Loading buffer), Lane 3: EQ5-HF (Total protein loading amount: 8 μg, with 2 µL 5 × Loading buffer), Lane 4: CNE-HF (Total protein loading amount: 8 μg, with 2 µL 5 × Loading buffer), Lane 5: IdeE-HF (Total protein loading amount: 8 μg, with 2 µL 5 × Loading buffer), Lane 6: EAG-HF (Total protein loading amount: 8 μg, with 2 µL 5 × Loading buffer).

**Figure 5:**
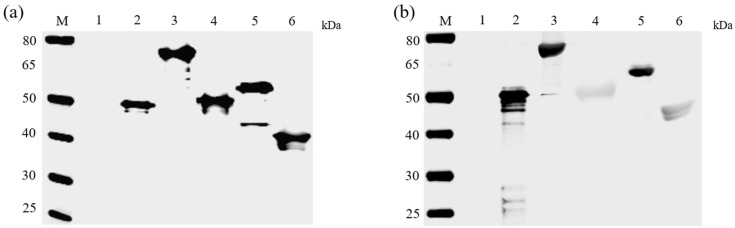
Western blot identification of five recombinant HF-fusion proteins. M: Protein marker. (**a**) Probed with anti-His antibody. Lane 1: PET28a empty vector (operation and loading amount were consistent with Lane 1 in Figure 4), 2: EQ8-HF (Total protein loading amount: 8 μg, with 2 µL 5 × Loading buffer), Lane 3: EQ5-HF (Total protein loading amount: 8 μg, with 2 µL 5 × Loading buffer), Lane 4: CNE-HF (Total protein loading amount: 8 μg, with 2 µL 5 × Loading buffer), Lane 5: IdeE-HF (Total protein loading amount: 8 μg, with 2 µL 5 × Loading buffer), Lane 6: EAG-HF (Total protein loading amount: 8 μg, with 2 µL 5 × Loading buffer). (**b**) Probed with positive equine serum. The samples and loading amounts of Lane 1 to Lane 6 were exactly the same as those in Figure 5a.

The theoretical molecular weights of the five recombinant proteins are: rEQ8-HF:49 kDa, rEQ5-HF: 73 kDa, rCNE-HF: 54 kDa, rIdeE-HF: 56 kDa, and rEAG-HF: 43 kDa. All five recombinant fusion proteins were successfully expressed and purified (Figure 4). The five fusion proteins were divided into two parallel groups for Western blotting analysis. All of these proteins could be specifically recognized by the His-tag antibody (Figure 5a) and positive equine serum (Figure 5b), the faint band of CNE-HF may result from its low immunodominance in natural equine infection and partial epitope masking in the fusion protein, as previously reported for multi-component *S. equi* subunit vaccines [32].

In summary, Western blot analysis confirmed that the five recombinant proteins exhibited favorable immunogenicity and retained intact native antigenic epitopes [33].

### 3.3. TEM and DLS Analysis

**Figure 6:**
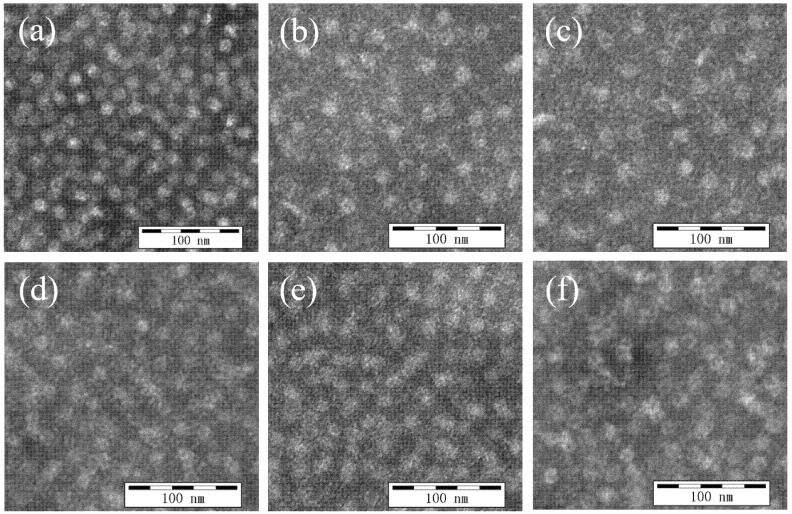
TEM observation of rHF and five recombinant fusion proteins. (**a**) rHF; (**b**) rEQ8-HF; (**c**) rEQ5-HF; (**d**) rCNE-HF; (**e**) rIdeE-HF; (**f**) rEAG-HF (scale bar: 100 nm).

TEM (Figure 6) revealed that purified HF assembled into spherical nanoparticles of approximately 10 nm. consistent with structural predictions, confirming correct assembly after expression and purification. The five recombinant fusion proteins (rEQ8-HF, rEQ5-HF, rCNE-HF, rIdeE-HF, rEAG-HF) also exhibited spherical structures, demonstrating successful antigen fusion without disrupting ferritin’s cage-like nanocarrier structure.

**Figure 7:**
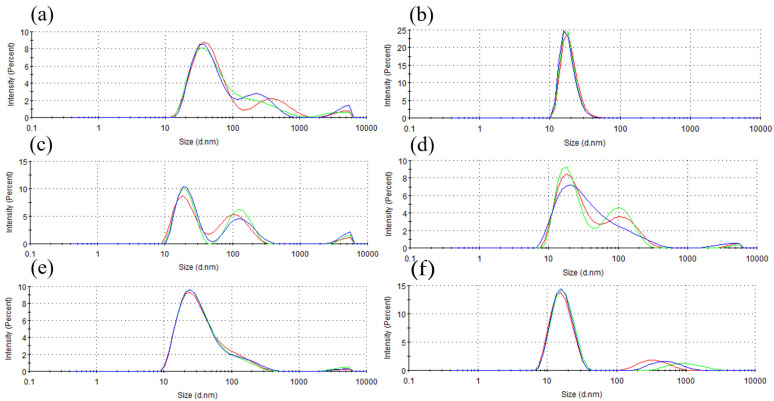
DLS analysis of rHF and five recombinant fusion proteins. (**a**) rEQ8-HF; (**b**) rEQ5-HF; (**c**) rCNE-HF; (**d**) rIdeE-HF; (**e**) rEAG-HF; (**f**) rHF. Red, green and blue lines represent three technical replicates of DLS measurement.

DLS analysis (Figure 7, Table 3) demonstrated that rHF displayed an average hydrated particle size of 17.27 nm. As DLS determines particle dimensions according to light scattering signals in liquid phase, it measures the hydrated size of protein nanoparticles in aqueous solution (including the water layer attached to the protein surface) [34], rather than the real physical diameter observed by TEM under dry conditions [35]. Therefore, the relatively larger value obtained by DLS represents a typical feature of this method and is in accordance with earlier publications.

TEM and DLS confirmed that rHF and the five fusion proteins correctly self-assembled into uniform spherical nanoparticles.

### 3.4. Protective Immunity Assay Against rSE5Mix

In the protective immunity assay, all rSE5Mix-immunized mice achieved complete protection, while all rHF-immunized and sterile PBS control mice died within 7 days post-challenge (Figure S1a), confirming rSE5Mix’s full protective efficacy and HF’s lack of immunogenicity alone. Body weight of rSE5Mix group mice began recovering on day 4 post-challenge and returned to pre-challenge levels by day 7, whereas rHF and control groups showed rapid, unrelenting weight loss (Figure S1b). Specific antibodies against the five antigens were detectable at week 1 post-primary immunization, rose continuously to peak at week 4, and remained stable thereafter (Figure S2). Normality and variance homogeneity were confirmed prior to statistical analysis.

### 3.5. Comparative Protective Immunity Assay of rSE5Mix and rSE8

**Figure 8:**
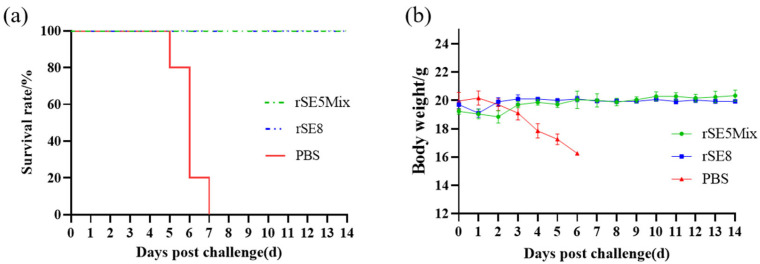
Mouse survival rate (**a**) and body weight changes (**b**) after challenge. Data are presented as mean ± SD (*n* = 10 mice per group). Normality and variance homogeneity were confirmed prior to statistical analysis.

In the comparative immunization trial of rSE5Mix and rSE8, survival and body weight changes of mice were continuously observed and recorded for 2 weeks after challenge. The results (Figure 8a) indicated that both rSE5Mix and rSE8 provided satisfactory protective effects, achieving full survival in mice after challenge.

Body weight monitoring (Figure 8b) revealed that the body weight of mice in the rSE8 group returned to normal on day 2 post-challenge. The average body weight of mice in the rSE5Mix group decreased after challenge, began to rebound on day 2 post-challenge, and recovered to the pre-immunization state by day 4. Both rSE5Mix and rSE8 conferred complete protection against lethal challenge with *S. equi*, with normal body weight recovery. Normality and variance homogeneity were confirmed prior to statistical analysis.

### 3.6. Specific Antibody Responses

Protective immunity against *S. equi* largely depends on the action of antigen-specific antibodies. Therefore, we monitored the post-immunization time course of antigen-specific IgG titers.

**Figure 9:**
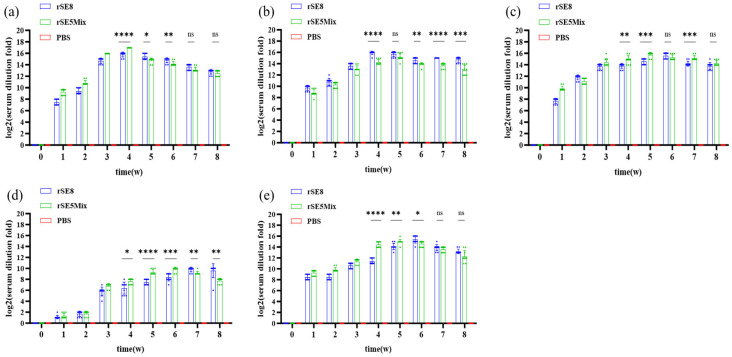
Antigen-specific antibody responses in mouse serum detected by indirect ELISA. (**a**) An-ti EQ8 antibody; (**b**) Anti EQ5 antibody; (**c**) Anti CNE antibody; (**d**) Anti IdeE antibody; (**e**) Anti EAG antibody. The *x*-axis denotes weeks post primary immunization, and the *y*-axis shows specific antibody titers presented as log_2_ serum dilution folds. All data were analyzed and plotted with GraphPad Prism 8.0. Data are presented as mean ± SEM (*n* = 10 per group). Normality and variance homogeneity were confirmed prior to statistical analysis. Two-way ANOVA with repeated measures followed by Tukey’s multiple comparisons test was used for statistical analysis. * *p* < 0.05, ** *p* < 0.01, *** *p* < 0.001, **** *p* < 0.0001, ns, not significant.

The optical density at 450 nm (OD_450nm_) was measured using a microplate reader. The cut-off value for each antigen was defined as the mean OD value of negative serum samples plus three standard deviations (mean + 3SD). Thresholds for positive antibody signals were determined in our previous study as follows: EQ8 = 0.08, EQ5 = 0.14, CNE = 0.13, IdeE = 0.13, and EAG = 0.21 [25]. Serum samples were subjected to two-fold serial dilution to measure log_2_-transformed antibody titers (Figure 9).

As shown in Figure 9, at one week post-primary immunization (1 wpi), the rSE5Mix group induced significantly higher titers against EQ8, CNE, IdeE, and EAG compared to the rSE8 group, demonstrating that the multi-valent nanoparticle vaccine initiates immune responses more rapidly.

Furthermore, the peak antibody titers of both the rSE5Mix and rSE8 groups reached the level of log_2_16. Compared with rSE8, rSE5Mix could elicit a more rapid specific antibody response against EQ8 and CNE in vivo.

### 3.7. Cytokine Expression

**Figure 10:**
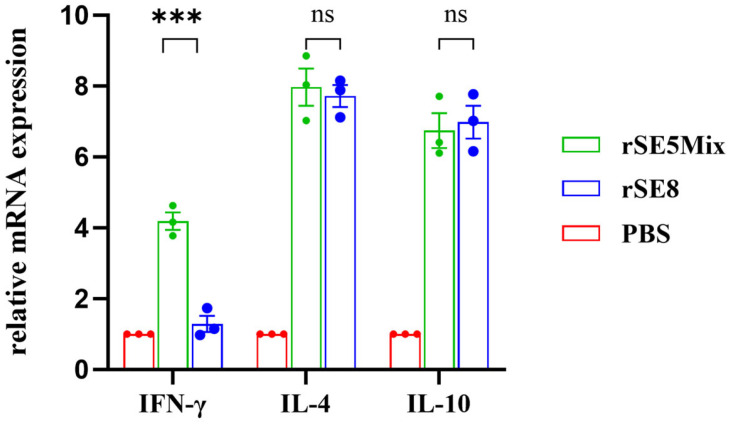
mRNA expression levels of IL-4, IL-10, and IFN-γ in mouse spleens detected by qRT-PCR. All experimental data were analyzed using GraphPad Prism 8.0. The Shapiro-Wilk test was performed for normality evaluation. Data are presented as mean ± SEM (*n* = 3 per group). *** *p* < 0.001.

The expression patterns of IL-4, IL-10 and IFN-γ can reflect the immune response type induced by vaccines, and the transcriptional levels of the three cytokines in mouse spleen tissues were detected at 24 h post challenge(Figure 10); rSE5Mix showed superiority in upregulating IL-4, IFN-γ and IL-10 expression, the significantly elevated levels of IL-4 and IL-10 compared with the control group indicated that rSE5Mix mainly triggered humoral immune responses and effectively alleviated inflammatory reactions in mice, while the IFN-γ expression level induced by rSE5Mix was higher than that in the rSE8 group.

### 3.8. Histopathology and Bacterial Load

**Figure 11:**
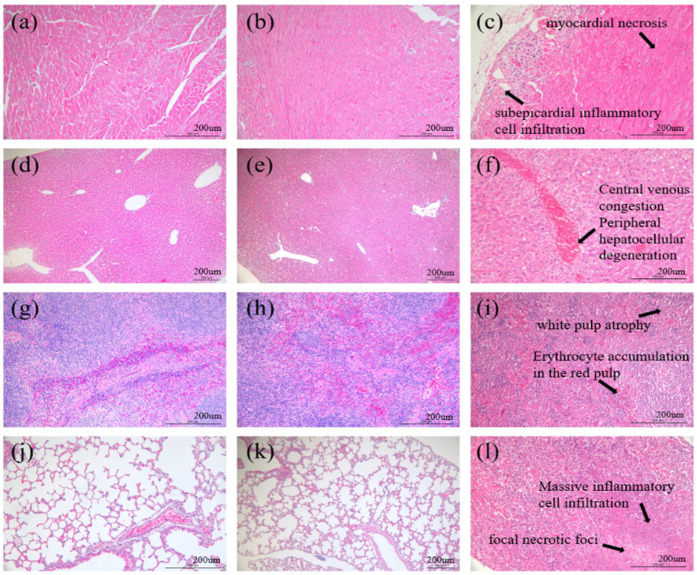
Pathological sections of mouse heart, liver, spleen, and lung. (**a**) Heart from the rSE5Mix group; (**b**) Heart from the rSE8 group; (**c**) Heart from the PBS control group; (**d**) Liver from the rSE5Mix group; (**e**) Liver from the rSE8 group; (**f**) Liver from the PBS control group; (**g**) Spleen from the rSE5Mix group; (**h**) Spleen from the rSE8 group; (**i**) Spleen from the PBS control group; (**j**) Lung from the rSE5Mix group; (**k**) Lung from the rSE8 group; (**l**) Lung from the PBS control group (scale bar: 200 μm).

Major organs including the heart, liver, spleen, and lung were collected from moribund mice. Tissue sections were prepared via fixation, paraffin embedding, and HE staining, followed by histomorphological observation under a light microscope. Mice in the rSE5Mix group (Figure 11a,d,g,j) and rSE8 group (Figure 11b,e,h,k) showed significantly milder histopathological lesions in major organs compared with the PBS control group, only mechanical damage caused by manipulation was observed. The control group exhibited typical pathological changes in all examined organs: inflammatory cell infiltration beneath the epicardium with myocardial necrosis in the heart (Figure 11c);central venous congestion and peripheral hepatocellular degeneration in the liver (Figure 11f);white pulp atrophy, reduced lymphocytes, erythrocyte accumulation in the red pulp, and myeloid hyperplasia in the spleen (Figure 11i);and focal necrotic foci in the pulmonary parenchyma with massive neutrophil infiltration in the alveolar cavities (Figure 11l).

**Figure 12:**
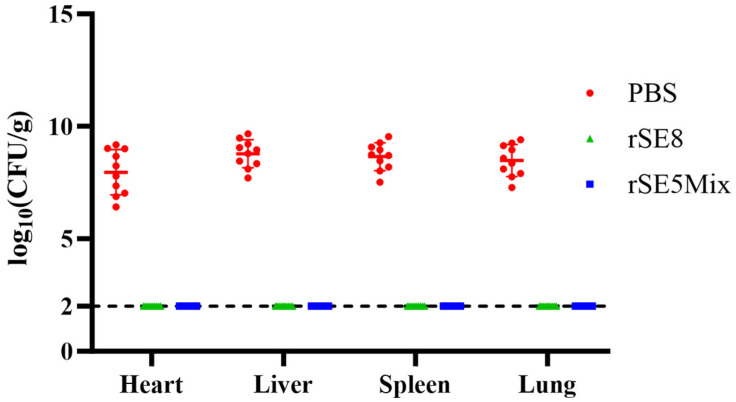
Bacterial loads in heart, liver, spleen, and lung of mice in each group. The dashed horizontal line indicates the limit of detection (LOD) of 10^2^ CFU/g. Bacterial counts below this line were considered undetectable. Samples with no detectable bacterial colonies were plotted at the detection limit. Data are presented as mean ± SD (*n* = 10 per group).

Bacterial isolation and identification results after challenge (Figure 12) indicated that the loads of pathogenic bacteria in heart, liver, spleen and lung tissues of mice in the rSE5Mix and rSE8 groups were all lower than the detection limit of 10^2^ CFU/mL, which was defined as nearly undetectable in this study. In accordance with relevant standards, bacterial concentrations ranging from 10^2^ to 10^3^ CFU/mL were regarded as low concentrations available for quantitative analysis, whereas concentrations below 10^2^ CFU/mL failed to be quantified [36]. By contrast, target pathogens were isolated from all four organs of mice in the control group, with bacterial loads of 6.3 × 10^7^ CFU/g in the heart, 2.9 × 10^8^ CFU/g in the liver, 5.58 × 10^8^ CFU/g in the spleen and 2.16 × 10^8^ CFU/g in the lung. Immunization with rSE5Mix or rSE8 could effectively eliminate invasive pathogens in vivo and alleviate visceral tissue damage.

### 3.9. Autoimmune Safety Evaluation of rHF in Horses

Serum-specific anti-GST-HF antibody levels were detected from pre-immunization to 4 weeks post-primary immunization. All horses remained clinically normal post-rHF immunization without obvious adverse reactions; mild transient hyperthermia in some horses resolved spontaneously within the physiological range (Figure S4).

ELISA results showed that anti-HF antibody levels were at basal levels before immunization, with no obvious inter-individual differences (Figure S5). Antibody levels increased after primary immunization, remained elevated despite slight fluctuations during the following weeks, and reached the highest levels after booster immunization. Although individual variation was observed among horses, anti-HF antibody levels generally remained above pre-immunization levels throughout the observation period.

As an equine homologous protein, rHF induced specific antibodies after two high-dose immunizations without clinical side effects, confirming its good safety in horses.

## 4. Discussion

The intrinsic adjuvant activity of equine ferritin-based nanoparticles arises from their orderly, multivalent antigen display, which promotes dendritic cell recognition and internalization and activates both antibody- and cell-mediated immunity without added adjuvants [4,5,6]. Strangles constitutes a severe and highly transmissible respiratory disorder in equines triggered by *S. equi*, which colonizes mucosal surfaces and lymphoid tissues [24,26,37], causing acute inflammation, abscess formation, and persistent economic losses to the global equine industry [27,38]. Traditional vaccines—such as inactivated whole-cell or live-attenuated preparations—continue to have limitations, including low immunogenicity, incomplete protection, and safety risks [20]. Therefore, the development of safe, effective, and fast-responding nanovaccines is of great significance for the efficient prevention and control of this disease [18,39].

In this study, a nanoparticle vaccine rSE5Mix based on recombinant equine ferritin (rHF) was constructed, displaying five protective antigens including EQ8, EQ5, CNE, IdeE and EAG. Using BALB/c mice as the animal model, the immunogenicity and protective efficacy of rSE5Mix were evaluated and compared with those of the rSE8 tandem fusion vaccine without additional adjuvants. Key experimental variables, such as the challenge dose of 10^6^ CFU, immunization procedure and feeding environment, were strictly controlled throughout the study. Standardized random grouping and single-blinded operations were adopted to reduce systematic errors. All these measures ensured the stability of experimental data, and the scatter plots also intuitively confirmed that individual differences were within a reasonable biological variation range. Protection against S. equi largely relies on humoral immune responses typified by high-affinity, high-titer antigen-specific IgG, which promotes bacterial opsonization, neutralization, and clearance [40]. In line with this, both rSE5Mix and rSE8 induced strong IgG responses and offered full protection against the lethal dose challenge.

Cytokine analysis further revealed differences in the immune response patterns induced by the two vaccines. Compared with the PBS control group, both rSE5Mix and rSE8 significantly promoted the expression of IL-4 and IL-10, showing an obvious Th2-biased immune characteristic, which could effectively facilitate B cell activation, enhance antibody production and mitigate inflammatory tissue damage. Besides, no significant difference was observed between the two vaccine groups.

Histopathological examination and bacterial isolation further confirmed that mice immunized with rSE5Mix or rSE8 exhibited significantly alleviated organ lesions, and barely detectable *S. equi* in the heart, liver, spleen, and lung. In contrast, control mice showed severe inflammatory infiltration, tissue necrosis, and high bacterial loads, indicating systemic infection and pathological damage. These results demonstrate that both vaccines effectively reduce bacterial colonization, systemic spread, and subsequent pathogenic injury [41,42].

These murine experimental results fully demonstrate that rSE5Mix possesses promising translational application prospects in the prevention and control of equine strangles and could provide complete protection and achieve 100% survival rate in mice. Meanwhile, HF exerts no additional immune effects while presenting antigens and does not impair the immune efficacy of the vaccine.

Statistical analysis showed that rHF induced significant changes in specific antibody levels in immunized horses. Under this condition, none of the five rHF-vaccinated horses developed persistent fever, depression, inappetence or other abnormal clinical symptoms, with only transient, slight hyperthermia observed. These results confirm that rHF possesses good biosafety with low autoimmunogenicity and does not trigger obvious autoimmune responses. This finding provides an important basis for the application of rHF as a carrier in equine vaccines, and also verifies that the ferritin carrier does not interfere with the immunogenicity of target antigens. Further equine trials are needed to assess mucosal immunity and protective duration. Compared with commercial vaccines, our rSE5Mix is low-cost, easy to produce, and scalable for large-scale application.

This study has several inherent limitations. First, all immunological evaluations and protective efficacy verifications were exclusively conducted in a mouse model. *Streptococcus equi* exhibits strict host specificity, with natural infections restricted solely to Equidae [43]; mice are not its natural susceptible hosts and merely serve for preliminary exploration and screening of vaccine immunogenicity. Although rSE5Mix provided complete protection against lethal-dose bacterial challenge in mice, this outcome may be associated with the intrinsic immune response characteristics of the mouse model. The mouse model cannot fully recapitulate the natural course of infection, mucosal immune features, pathological damage patterns, or the complex immune regulatory network observed in horses following natural *S. equi* infection. Therefore, the immune responses, organ pathological damage, and survival protection effects obtained in mice only offer limited references for clinical translation in natural hosts. The conclusions of this study should not be overextended, nor can they be directly extrapolated to practical clinical applications in horses.

The current equine trial was only a preliminary observation on safety and immunogenicity with a limited sample size and no independent control group, which can only serve as a preliminary basis for evaluating the application potential of the ferritin carrier. In follow-up studies, we will expand the number of experimental horses, set up additional control groups, prolong the observation period, and conduct systematic immunological assessment and protective efficacy evaluation to further optimize and complete the experimental verification system in the target natural host.

Second, the specific mechanisms by which this nanoparticle vaccine regulates immune cell subset differentiation, mediates immune memory formation, and maintains long-term immune persistence have not been fully elucidated. Third, only a single strain was used for challenge testing in this study; the broad-spectrum protective efficacy of rSE5Mix against different clinical isolates and prevalent strains awaits further in-depth investigation [44].

## 5. Conclusions

In conclusion, the equine ferritin-based nanoparticle vaccine rSE5Mix elicits rapid and strong humoral immune responses in mice, characterized by early antibody production, high titers, and specific responses against key protective antigens. Both rSE5Mix and rSE8 provide effective protection against lethal challenge with *S. equi*, accompanied by significantly alleviated histopathological lesions and reduced bacterial loads in major organs. Compared with rSE8, rSE5Mix shows advantages in the speed of antibody induction against several antigens. These results demonstrate that the equine ferritin nanoparticle platform exhibits favorable safety and immune presentation ability, representing a promising strategy for the development of novel vaccines against equine strangles.

## Figures and Tables

**Table 1:** The sequences for SOE-PCR.

Target Genes	Primer Sequences	Product Size (bp)
*eq8*	F: GCGACTACCCTAGCAGGACAAA	588
	R: GTGCTTAAGCTTTTCAATCTGA	
*eq5*	F: GAAACGACTACTGCTAGTGCAT	1320
	R: AGTAGCAACCAAGGCTGC	
*cne*	F: ACTAATCTTAGTGACAACATCA	912
	R: CTTGACAGTAAAGCTGGTATAG	
*idee*	F: GACGACTACCAGCGTAAC	945
	R: AGAGAGCTTCTGCCAGATGTCT	
*eag*	F: CTGGATGCTGCGACCGTT	483
	R: TTTAGCCTTGTTGATCAAGGTAA	

**Table 2:** The sequences for qRT-PCR.

Target Genes	Primer Sequences	Product Size (bp)
IL-10	F: ACAGCCGGGAAGACAAT	66
	R: GCAGCTCTAGGAGCATGT	
IL-4	F: TCCTGCTCTTCTTTCTCG	98
	R: TTTTCCTGTGACCTCGTT	
IFN-γ	F: CGCTACACACTGCATCTTGG	130
	R: TTCCACATCTATGCCACTTGAG	
β-actin	F: GAGACCTTCAACACCCCAGCC	263
	R: AATGTCACGCACGATTTCCC	

**Table 3:** Average particle size from DLS analysis of five recombinant proteins and rHF.

Protein Name	rEQ8-HF	rEQ5-HF	rCNE-HF	rIdeE-HF	rEAG-HF
Average particle size/nm	51.91	36.93	32.72	26.81	26.82

## Data Availability

The original contributions presented in this study are included in the article/Supplementary Materials. Further inquiries can be directed to the corresponding authors.

## Supplementary Materials

The following supporting information can be downloaded at https://www.mdpi.com/article/10.3390/vetsci13060527/s1, Figure S1: Mouse survival rate (a) and body weight changes (b) after challenge.; Figure S2: Antigen-specific antibody responses in mouse serum detected by indirect ELISA.; Figure S3: SDS-PAGE of six GST-tagged proteins. Figure S4: Changes in horse body temperature within 5 days after rHF immunizations. Figure S5: Specific antibody levels (OD values) against HF in horse serum within 4 weeks after the first immunization with recombinant equine ferritin (rHF).

## Abbreviations

rHF	recombinant equine ferritin
rSE5Mix	five recombinant antigen mixed ferritin nanoparticle vaccine
rSE8	recombinant eight-antigen tandem ferritin vaccine
TEM	transmission electron microscopy
DLS	dynamic light scattering
iELISA	indirect enzyme-linked immunosorbent assay
qRT-PCR	quantitative real-time polymerase chain reaction
IL-4	interleukin-4
IL-10	interleukin-10
IFN-γ	interferon-gamma
LOD	limit of quantification
Th1	T helper type 1
Th2	T helper type 2
CFU	colony-forming unit
MLD	minimum lethal dose
PBS	phosphate-buffered saline
SDS-PAGE	sodium dodecyl sulfate–polyacrylamide gel electrophoresis
