# Supplementary material for: Development and Immunogenicity of a Five-Antigen Strangles Vaccine Based on Equine Ferritin Nanoparticles in Mice
Source: Vet Sci. 2026 May 28;13(6):527. doi: 10.3390/vetsci13060527 (PMC13307852; doi:10.3390/vetsci13060527)
Supplement: Supplementary file 1 [file vetsci-13-00527-s001.zip › vetsci-4296214-supplementary.pdf]

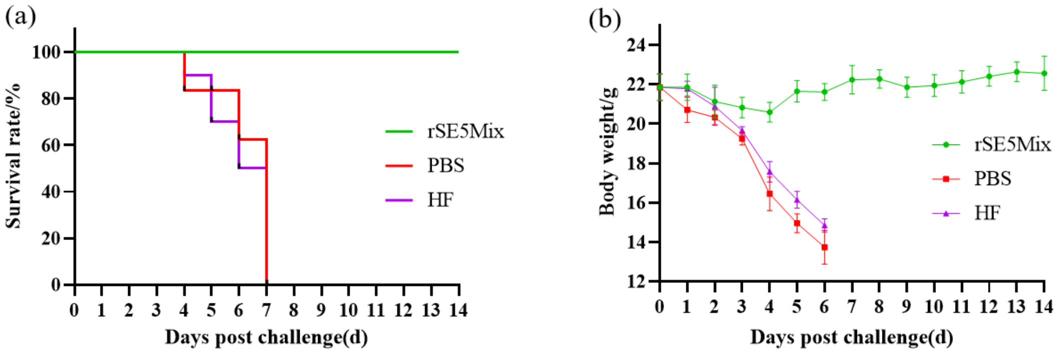

Figure S1. Mouse survival rate (a) and body weight changes (b) after challenge. Data are presented as mean ± SD (n = 10 mice per group). Normality and variance homogeneity were confirmed prior to statistical analysis.

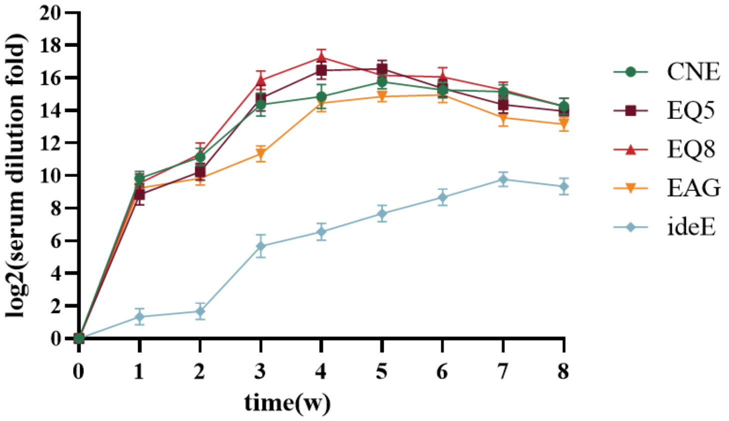

Figure S2. Antigen-specific antibody responses in mouse serum detected by indirect ELISA. The x-axis represents weeks after primary immunization, and the y-axis shows specific antibody titers presented as $\log_2$ (serum dilution folds). All data were analyzed and plotted using GraphPad Prism 8.0 software. Data are presented as mean ± SEM (n = 10 per group).

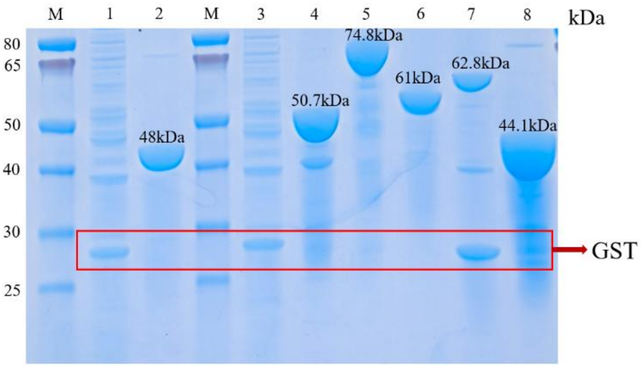

Figure S3.SDS-PAGE of six GST-tagged proteins. M: Protein molecular weight marker; Lane 1: pGEX-6p-1 empty vector; Lane 2: GST-HF; Lane 3: Empty vector; Lane 4: GST-EQ8; Lane 5: GST-EQ5; Lane 6:

GST-CNE; Lane 7: GST-IdeE; Lane 8: GST-EAG.

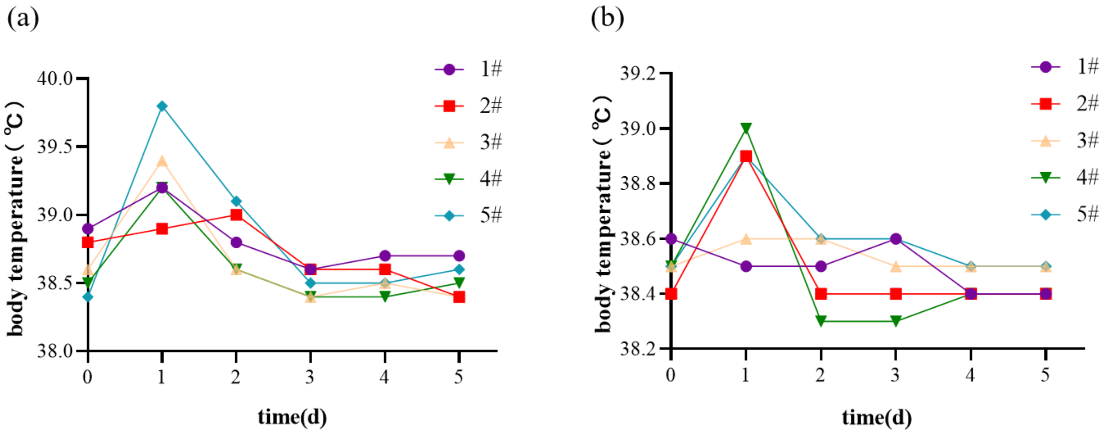

Figure S4. Changes in horse body temperature within 5 days after rHF immunizations. (a) shows the changes in horse body temperature from day 0 to day 5 after the first immunization with rHF protein; (b) shows the changes in horse body temperature from day 0 to day 5 after the second immunization with rHF protein

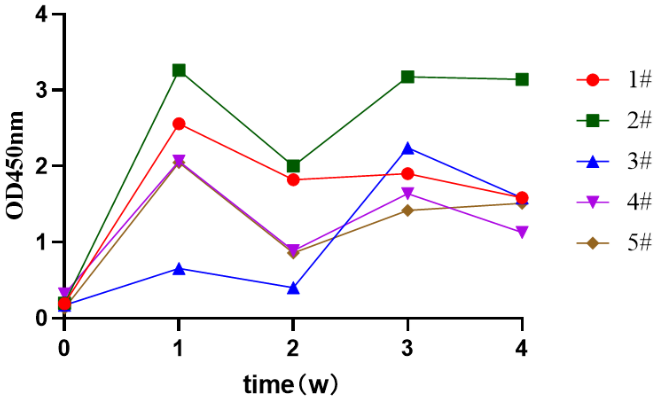

Figure S5. Specific antibody levels (OD values) against HF in horse serum within 4 weeks after the first immunization with recombinant equine ferritin (rHF).
