# Supplementary material for: Development and Immunogenicity of a Five-Antigen Strangles Vaccine Based on Equine Ferritin Nanoparticles in Mice
Source: Vet Sci. 2026 May 28;13(6):527. doi: 10.3390/vetsci13060527 (PMC13307852; doi:10.3390/vetsci13060527)
Supplement: Supplementary file 1 [file vetsci-13-00527-s001.zip › vetsci-4296214- WB.pdf]

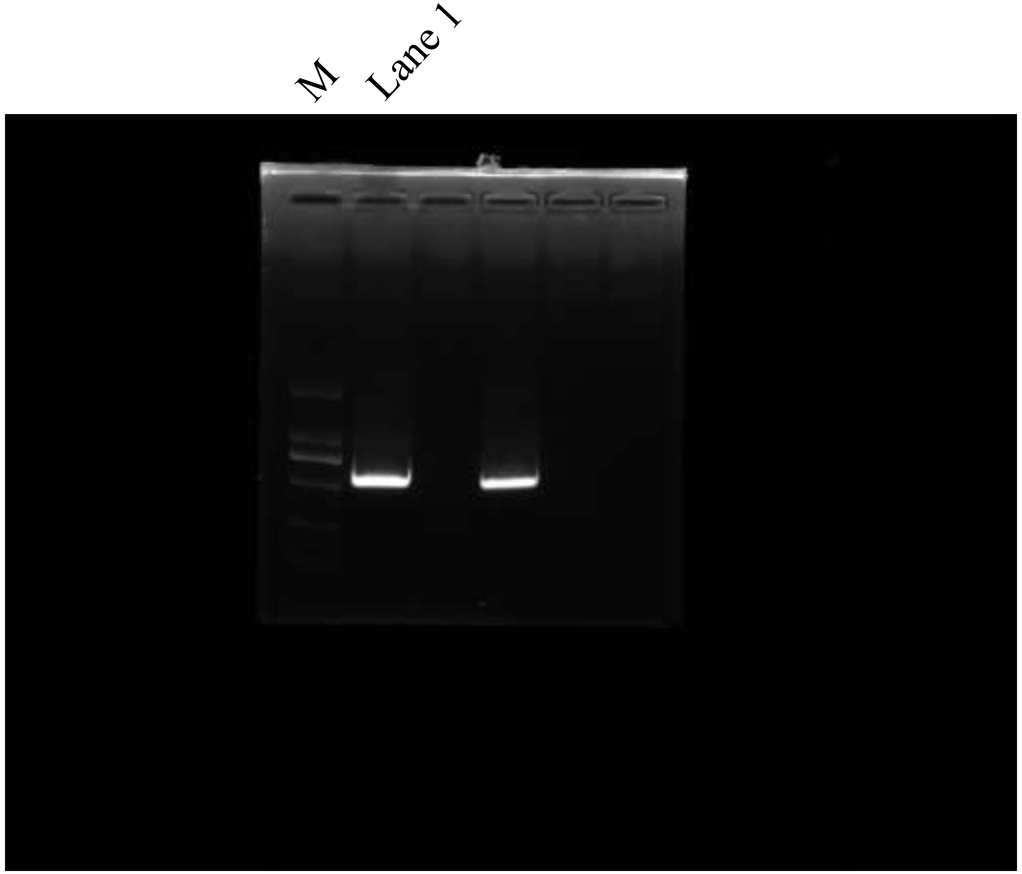

Figure 3a M: DNA marker; Lane 1: PCR product of HF.

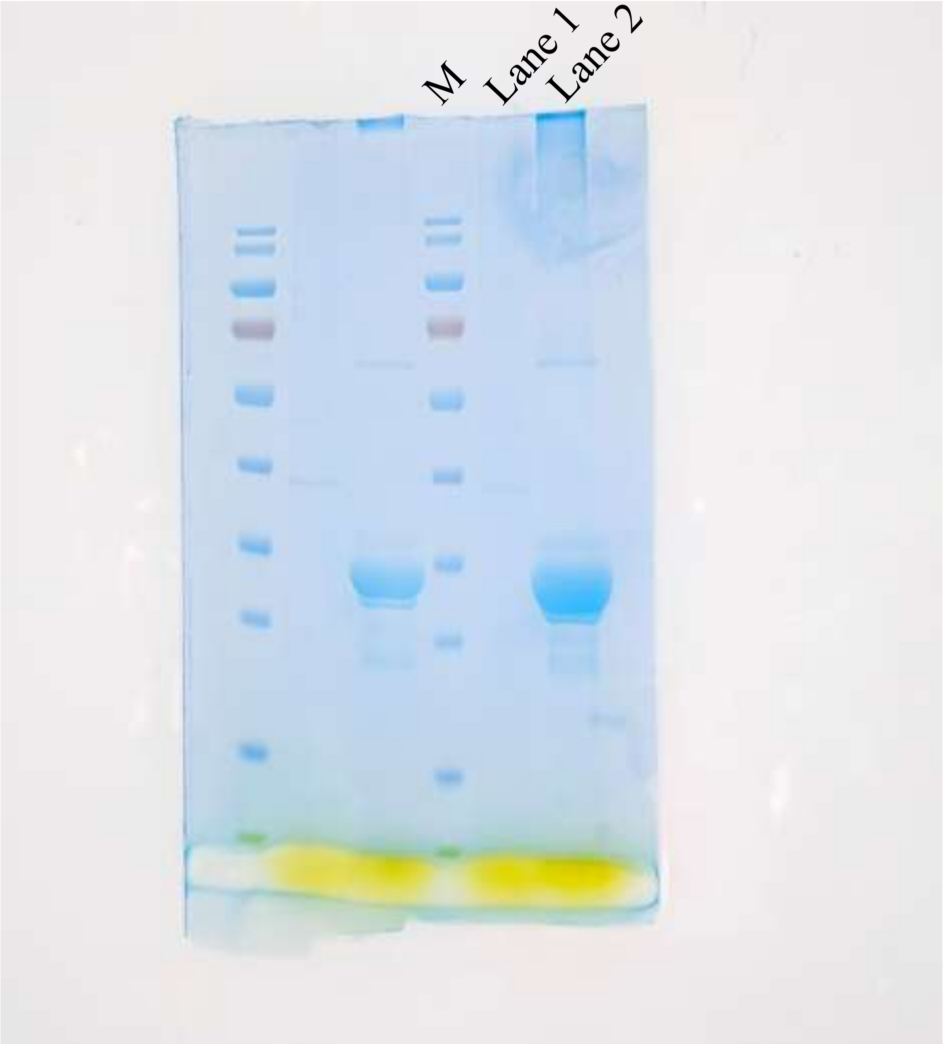

Figure 3b M: Protein marker; Lane 1: pET30a Empty vector; Lane 2: Purified rHF.

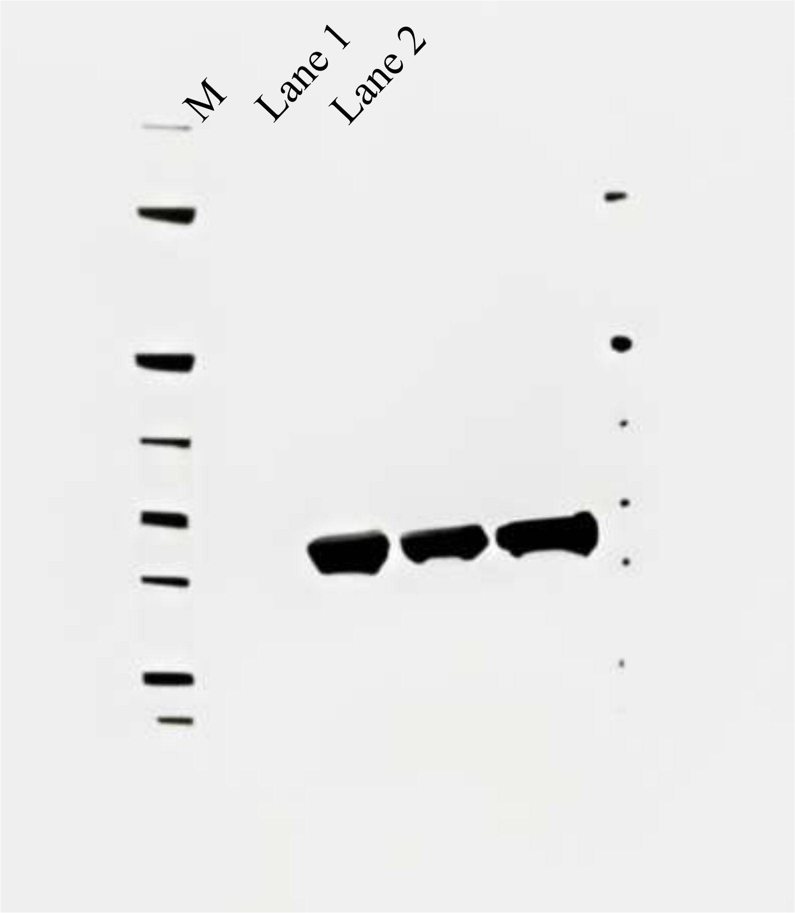

Figure 3c M: Protein marker; Lane 1: pET30a Empty vector; Lane 2: Purified rHF.

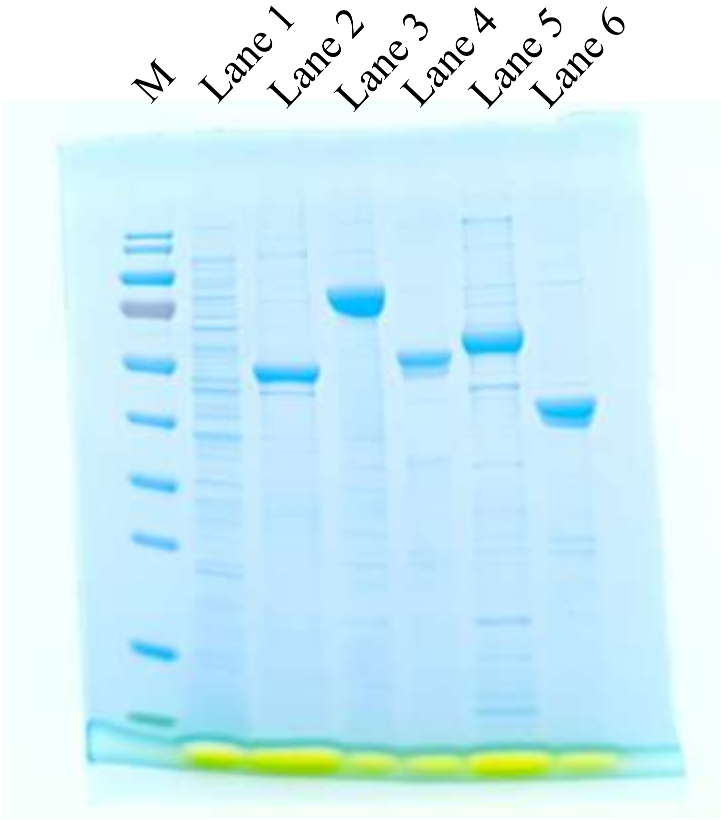

Figure 4.M: Protein marker. Lane 1: pET28a empty vector,Lane 2: EQ8-HF, Lane 3: EQ5-HF, Lane 4: CNE-HF, Lane 5: IdeE-HF, Lane 6: EAG-HF.

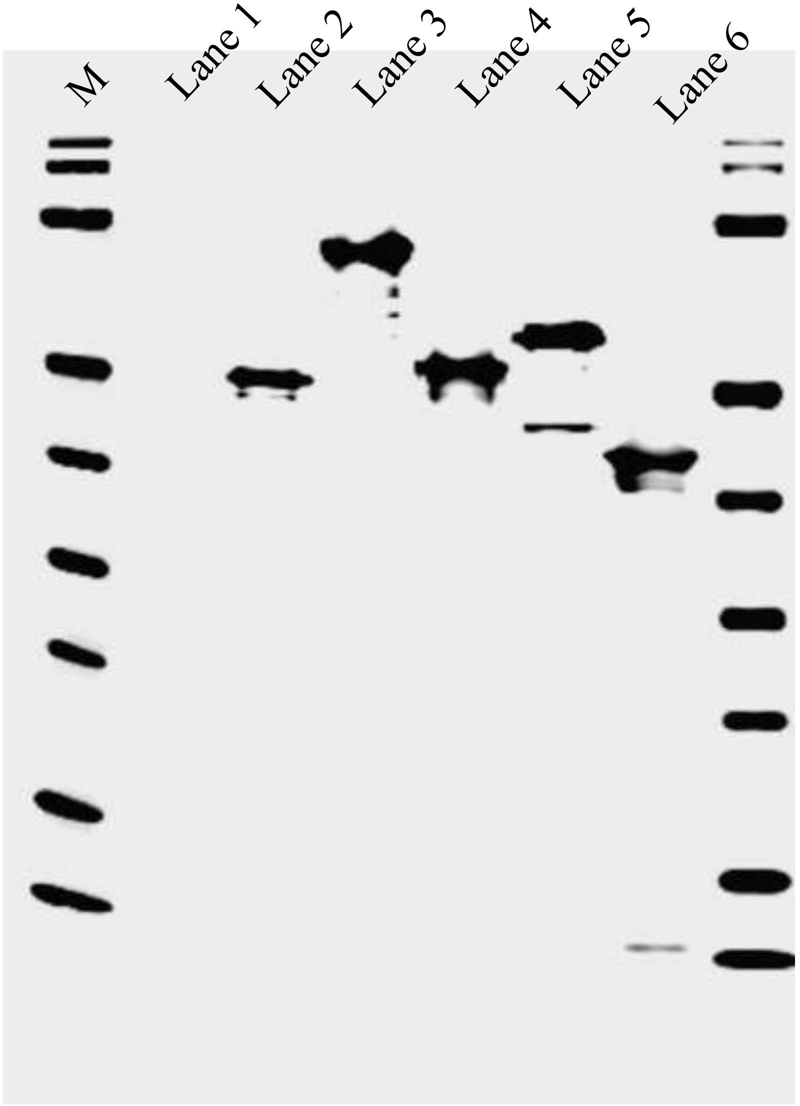

Figure 5a Protein marker. Lane 1: pET28a empty vector,Lane 2: EQ8-HF, Lane 3: EQ5-HF, Lane 4: CNE-HF, Lane 5: IdeE-HF, Lane 6: EAG-HF.

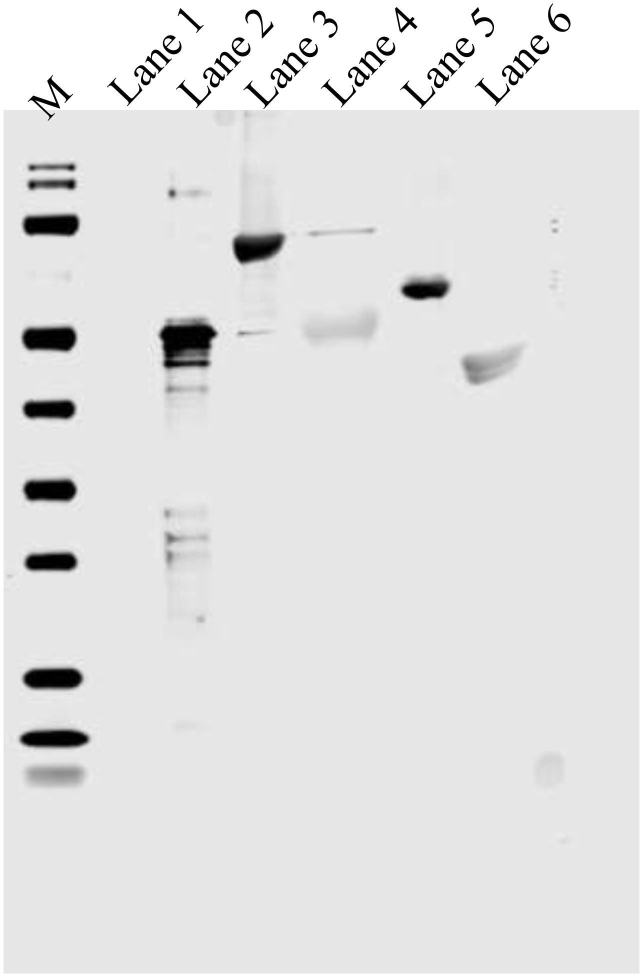

Figure 5b Protein marker. Lane 1: pET28a empty vector,Lane 2: EQ8-HF, Lane 3: EQ5-HF, Lane 4: CNE-HF, Lane 5: IdeE-HF, Lane 6: EAG-HF.

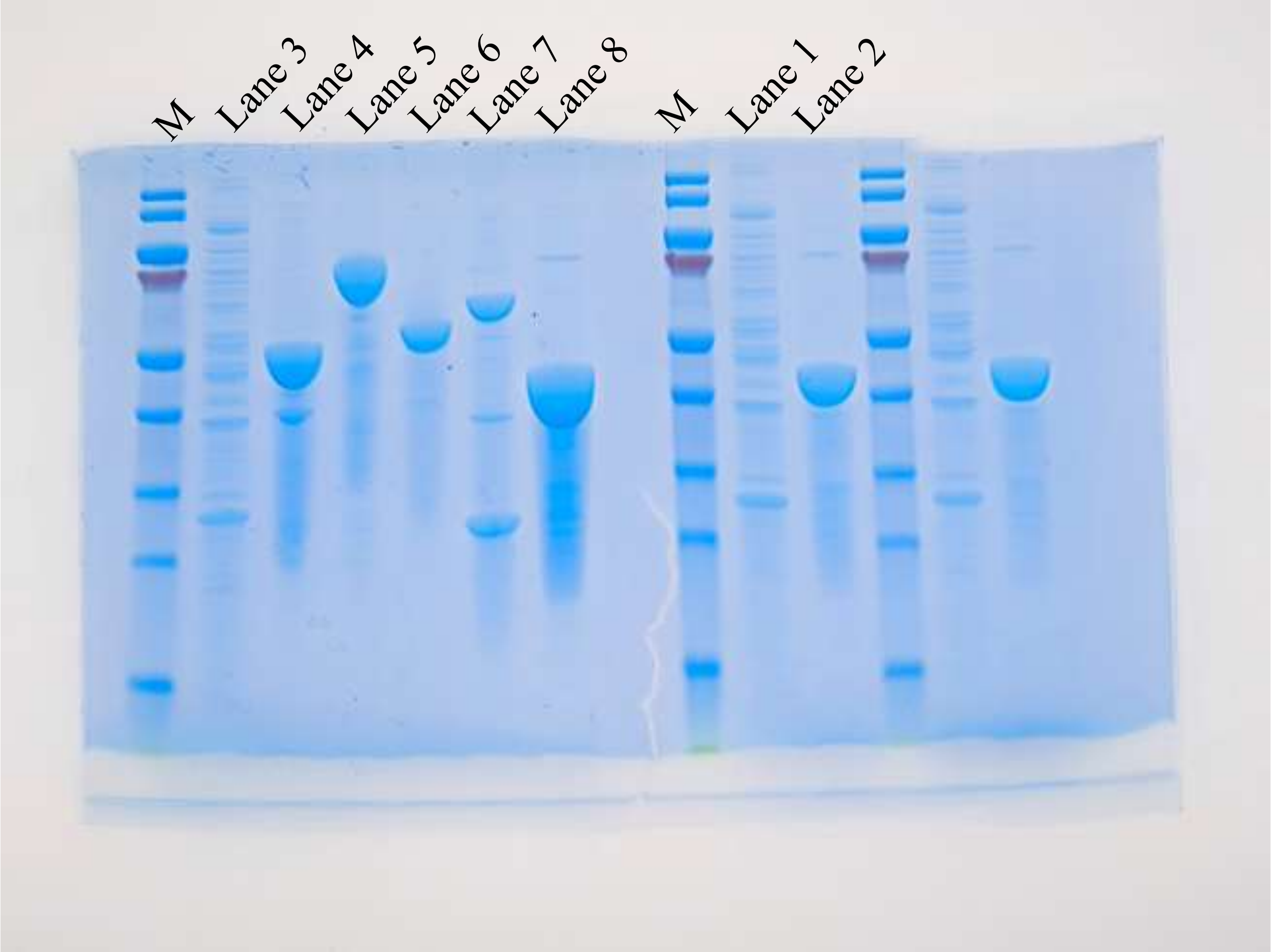

Figure S3. M: Protein molecular weight marker; Lane 1: pGEX-6p-1 empty vector; Lane 2: GST-HF; Lane 3: Empty vector; Lane 4: GST-EQ8; Lane 5: GST-EQ5; Lane 6: GST-CNE; Lane 7: GST-IdeE; Lane 8: GST-EAG.
